# Interacting effects of vessel noise and shallow river depth elevate metabolic stress in Ganges river dolphins

**DOI:** 10.1038/s41598-019-51664-1

**Published:** 2019-10-28

**Authors:** Mayukh Dey, Jagdish Krishnaswamy, Tadamichi Morisaka, Nachiket Kelkar

**Affiliations:** 1 P.G. Program in Wildlife Biology and Conservation, National Centre for Biological Sciences-TIFR, GKVK Campus, Bellary Road, Bangalore, 560065 Karnataka India; 2 Ashoka Trust for Research in Ecology and the Environment (ATREE), Royal Enclave Srirampura, Jakkur PO, Bangalore, 560064 Karnataka India; 3 Mie University, 1577 Kurimamachiya-cho, Mie, 514-8507 Japan

**Keywords:** Behavioural ecology, Conservation biology, Ecological modelling, Freshwater ecology, Ecology, Hydrology

## Abstract

In riverine ‘soundscapes’, complex interactions between sound, substrate type, and depth create difficulties in assessing impacts of anthropogenic noise pollution on freshwater fauna. Underwater noise from vessels can negatively affect endangered Ganges river dolphins (*Platanista gangetica*), which are ‘almost blind’ and rely entirely on high-frequency echolocation clicks to sense their environment. We conducted field-based acoustic recordings and modelling to assess acoustic responses of *Platanista* to underwater noise exposure from vessels in the Ganga River (India), which is now being transformed into a major waterway. Dolphins showed enhanced activity during acute noise exposure and suppressed activity during chronic exposure. Increase in ambient noise levels altered dolphin acoustic responses, strongly masked echolocation clicks, and more than doubled metabolic stress. Noise impacts were further aggravated during dry-season river depth reduction. Maintaining ecological flows, downscaling of vessel traffic, and propeller modifications to reduce cavitation noise, could help mitigate noise impacts on Ganges river dolphins.

## Introduction

Anthropogenic noise pollution is now a global threat, pervading terrestrial and aquatic ecosystems worldwide^1^. With an increase in human-made noise in the environment, the natural “soundscapes” and sonic cues used by animal species have been perturbed^2^. In aquatic environments, where visual cues are limited^3^, most species depend on sound to process information crucial to survival, since sound travels further and faster underwater^4,5^. Underwater noise from vessel traffic, SONAR applications, dredging, pile-driving for construction, etc. could thus negatively affect aquatic animal species^6–8^.

Odontocetes (toothed whales and dolphins) and mysticetes (baleen whales) are especially susceptible to underwater noise^9^ as they depend almost entirely on pulsed and tonal sounds for long-distance communication, foraging, navigation, and sensing their environment^4^. Low-frequency acoustic signals emitted by mysticetes are prone to interference from similar low-frequency noise produced by ship engines^10–12^. Odontocetes emit a range of low-frequency to ultrasonic acoustic signals and have wide hearing ranges, making them sensitive to diverse anthropogenic sound frequencies^9,10^. Underwater noise from vessel SONAR or cavitation noise from propellers can displace cetaceans from preferred habitats^13^. Enhanced acoustic activity^14,15^ can induce changes in foraging and diving behaviour^16–19^. This can result in temporary hearing loss^20–22^, high metabolic energy expenditure, and corresponding increases in stress hormone levels^19,23–26^. Noise interference can further force cetaceans to modify their acoustic signals to avoid masking^27–30^. Resulting energetic costs may cause loss of opportunities for feeding and resting, and reduce fitness^10,31–34^. Altered behaviour could further lead to fine-scale behavioural shifts or disorientation, which could lead dolphins to closer proximity to fishing nets or vessel propellers. This can potentially aggravate collision risk or bycatch from net entanglement^35–37^, increasing mortality or injury^38^ at the individual and population levels^39,40^.

Most studies on the effect of underwater noise have been conducted on marine cetaceans, whose avoidance of noise is not limited by the availability of space^19,40^. This contrasts with dolphins inhabiting spatially restricted and depth-limited environments such as rivers, lakes, and estuaries, in which noise impacts are poorly understood. A general prediction is that underwater noise pollution will be higher in shallow rivers due to greater reflection from the bottom and sides, and longer persistence of sound^41^. Large rivers show strong seasonal variability in depth, flood-pulses, sediment fluxes, and temperature, which can influence the extent of impact of underwater noise on river dolphins in complex ways^5,14,18,42,43^. River flow reduction through regulation by upstream dams or barrages or during droughts could also limit the river space available to dolphins to avoid noise.

Impacts of underwater vessel noise and propeller hits caused significant mortality of the Chinese river dolphin or Baiji (*Lipotes vexillifer*) from the Yangtze River in the 1950s and 1960s^37,44^. In later decades, mortality of the remaining Baiji populations due to destructive fishing practices caused the species’ extinction^37,45^. Unfortunately, the exact mechanisms underlying the susceptibility of Baiji to underwater noise remain unknown. But its extinction offers context to test the prediction that underwater noise might impact South Asian river dolphins of the genus *Platanista* (endangered as per IUCN)^46^.

*Platanista* are obligate freshwater cetaceans that evolved about 24–26 million years ago (Ma) in the shallow, sediment-rich waters of the Indus-Ganges-Brahmaputra and associated river basins in the Indian sub-continent. They are ‘effectively blind’, with the crystalline lens of the eye and optic nerves having undergone evolutionary degeneration due to disuse in murky riverine habitats^47–49^. They also have the longest ear canals among extant dolphins, and show side swimming, both of which are highly specialized adaptations for life in shallow rivers^50^. These dolphins rely almost entirely on high-frequency echolocation clicks (55–75 kHz peak frequency) and are described as “continuous emitters”^51,52^, relying only on modulations of their highly directional clicks for communication^50,52^. Importantly, *Platanista* do not produce whistles, which are low-frequency sounds in other cetaceans that overlap closely with frequencies of anthropogenic underwater noise^53^. *Platanista* thus offers a unique context to study effects of anthropogenic noise on river dolphins in shallow riverine environments with seasonal flow variability.

The recent and proposed expansion of industrial transportation and recreational waterways in the Ganga-Brahmaputra river basins^54,55^ might expose *Platanista* to increasing underwater noise from vessels and river-bottom dredging^56^, in addition to pre-existing major threats, e.g. declining/altered river flows, fisheries bycatch mortality, targeted killing, poaching, and pollution^46,57^. Increased vessel traffic and associated maintenance dredging are expected to overlap nearly 90% of the current range of Ganges river dolphin populations^58^. This situation presents a timely opportunity to scientifically demonstrate the pathways, extent, nature, and magnitude of underwater noise impacts on the ecology and behaviour of *Platanista*.

Our study aimed to understand acoustic responses of Ganges river dolphins along a gradient of ambient underwater noise levels in the Ganga River, Bihar, India. At our reference site, we assessed underwater noise impacts along with river depth and biological variables (fishing pressure) on *Platanista*. We hypothesized that river dolphins would alter their echolocation click properties and acoustic activity from baseline levels, in the presence of underwater noise from motorised vessels. We predicted that *Platanista* will modify the level of acoustic activity (click-rates, train duration), loudness (sound pressure level), and click frequency to overcome constraints imposed by vessel induced noise (e.g. interference with hearing and masking of clicks). During chronic noise exposure, river dolphins would compensate for masking by enhancing their acoustic activity, but for acute/episodic exposure, would suppress acoustic activity. We expected greater underwater noise impacts in shallower channel conditions due to higher reverberation and persistence of noise in the water column.

We tested these predictions with passive acoustic monitoring of river dolphins and vessel noise in “quiet” (before-vessel passage) and “noisy” (during-vessel passage) phases, at different vessel traffic rates, and across a gradient of river depth and discharge. We then modelled masking ranges and metabolic costs (stress/energy demand) of altered acoustic activity for individual river dolphins under enhanced noise exposure, and estimated masking range for communicating river dolphins. Based on our results, we discuss (1) effects of metabolic stress on river dolphins; (2) the need to maintain river flow and depth to buffer impacts of noise; and (3) mitigation of risks from underwater noise impacts from inland waterways on endangered Ganges river dolphins in India.

## Results

### Change in ambient noise level and boat traffic at reference site (Nov 2017–Mar 2018)

**Figure 1:**
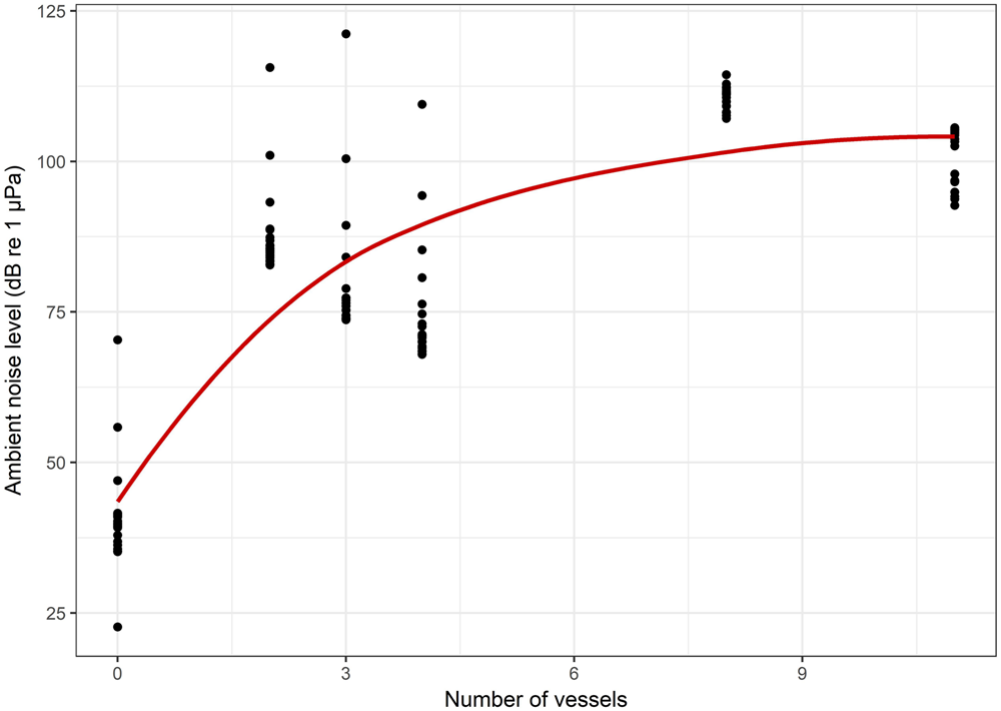
A non-linear relationship was found for the number of vessels plying and the corresponding ambient noise level. The highest degree of increase would occur until 4 to 5 vessels, beyond which the ambient noise level reached an asymptote.

Ambient underwater noise level increased non-linearly with increase in vessel movement (boats per hour) as the dry-season progressed (asymptote at 105.66 dB re 1 μPa, R_0_ = 43.44, natural logarithm of rate constant (LRC) = −1.08; p-value < 0.001; residual std. error = 12.28; df = 124). Ambient noise levels peaked beyond a traffic intensity of >5 boats/hour (Fig. 1). In this period, river discharge at the reference site (Kahalgaon) reduced from 9150 m^3^/s to 6430 m^3^/s over November-March, i.e. by almost 30% in a period of about 120 days. This resulted in a corresponding depth reduction of almost 1.5 m. In this period, river dolphin spots remained spatially stable (<100 m shift from recording location).

### Dolphin acoustic responses to ambient noise levels at reference site (over time)

**Figure 2:**
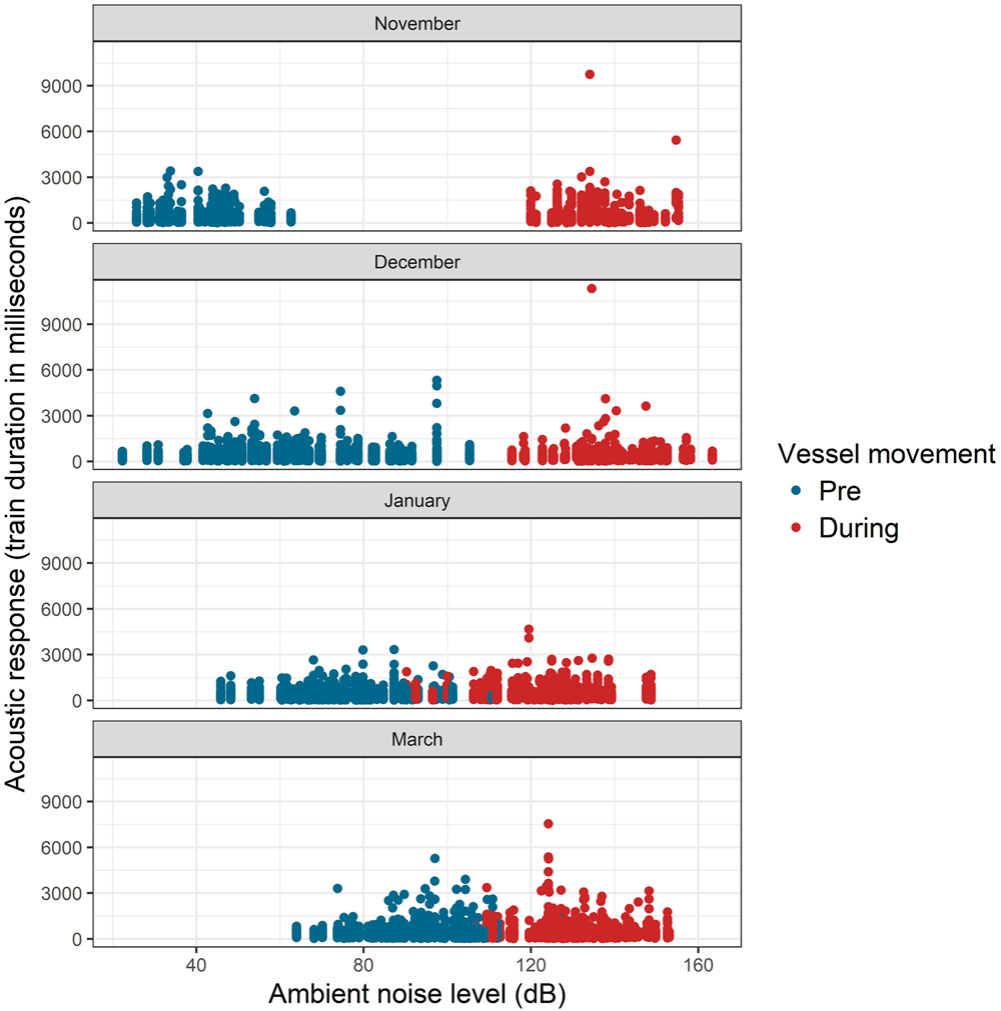
From November to March, as river discharge reduced, vessel traffic increased, and ambient noise levels increased, the magnitude of difference reduced substantially in the ‘pre’ and ‘during’ phases for an acoustic response (train duration) at the reference site (Kahalgaon).

**Figure 3:**
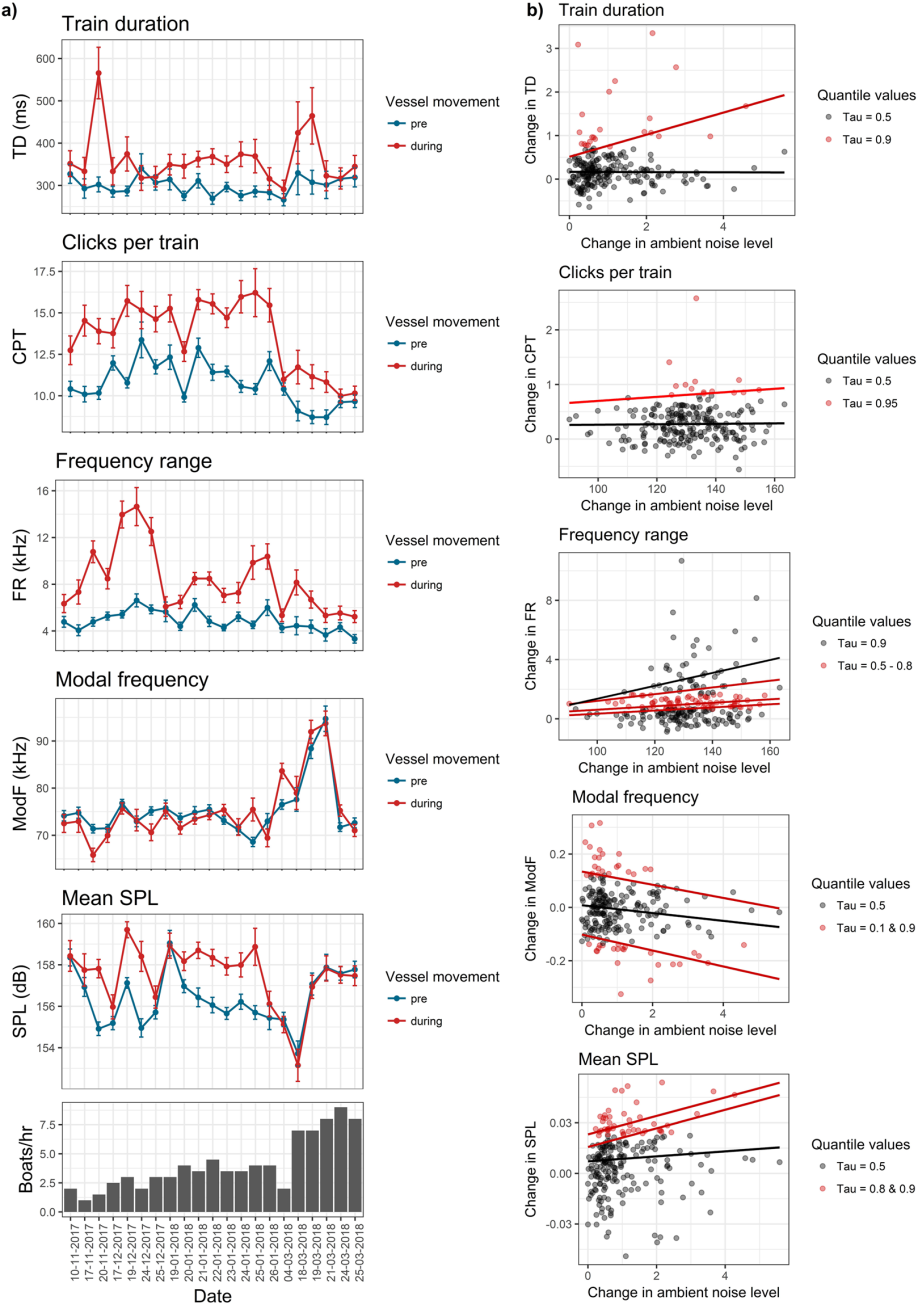
(**a**) Time-series graphs depicting dolphin responses to vessel noise as compared to baseline levels at the reference site (Kahalgaon). Means and 95% CI are plotted together for (i) Train duration; (ii) Clicks per train; (iii) Frequency range; (iv) Modal frequency; (v) Mean SPL, in relation to increase in number of vessels per hour from November 2018 to March 2019 (bottom panel). (**b**) Results of quantile regression models fitted to the five acoustic responses. Frequency and SPL values showed stronger responses to relative change in ambient noise levels than click rates or train duration (details in supporting information S5).

Ambient noise levels during the ‘pre-vessel’ phase increased in the river with the progress of the dry-season, as vessel traffic density increased from November to March. All acoustic responses of dolphins: train duration (TD), clicks per train (CPT), frequency range (FR), modal (peak) frequency (PF), and mean sound pressure level (SPL) in the ‘pre’ and ‘during’ phases became increasingly similar over this period (Fig. 2). Statistically significant differences were recorded for TD, CPT, and FR between the ‘pre’ and ‘during’ phases across recordings (Fig. 3a). Dolphins had consistently higher acoustic activity during acute noise exposure phases relative to baseline activity in quiet periods. However, PF and SPL showed complex responses. PF was significantly lower in the ‘during’ phase than baseline, when vessel traffic was lower in the early dry-season (November). PF was higher than baseline when vessel traffic was higher, in the peak dry-season (March) (Fig. 3a). From November to February, SPL was higher in ‘during’ phases relative to baseline ‘pre’ phases (Fig. 3a). But in March, when vessel traffic and noise increased substantially, SPL in the ‘during’ phase closely matched the baseline SPL in the ‘pre’ phase (Fig. 3a). These results contradicted our initial hypotheses about altered acoustic activity under acute and chronic noise exposure. Statistical comparisons of acoustic responses in pre-vessel and during-vessel phases are provided in Supplementary material S5 (Table S2).

### Effect of increasing underwater ambient noise on dolphin acoustic response

Quantile regression models indicated positive relationships between proportional change in ambient noise level and the proportional change in acoustic responses, although PF reduced with increase in vessel noise. The relationships were consistent and statistically significant for higher quantiles of response variables (*tau* = 0.6–0.9, Fig. 3b). Models with statistically significant parameter estimates for intercept and effect size/slope for the respective quantiles are shown in Table S3 in Supplementary material S5.

### Acoustic responses of river dolphins to noise levels and depths across sites

**Figure 4:**
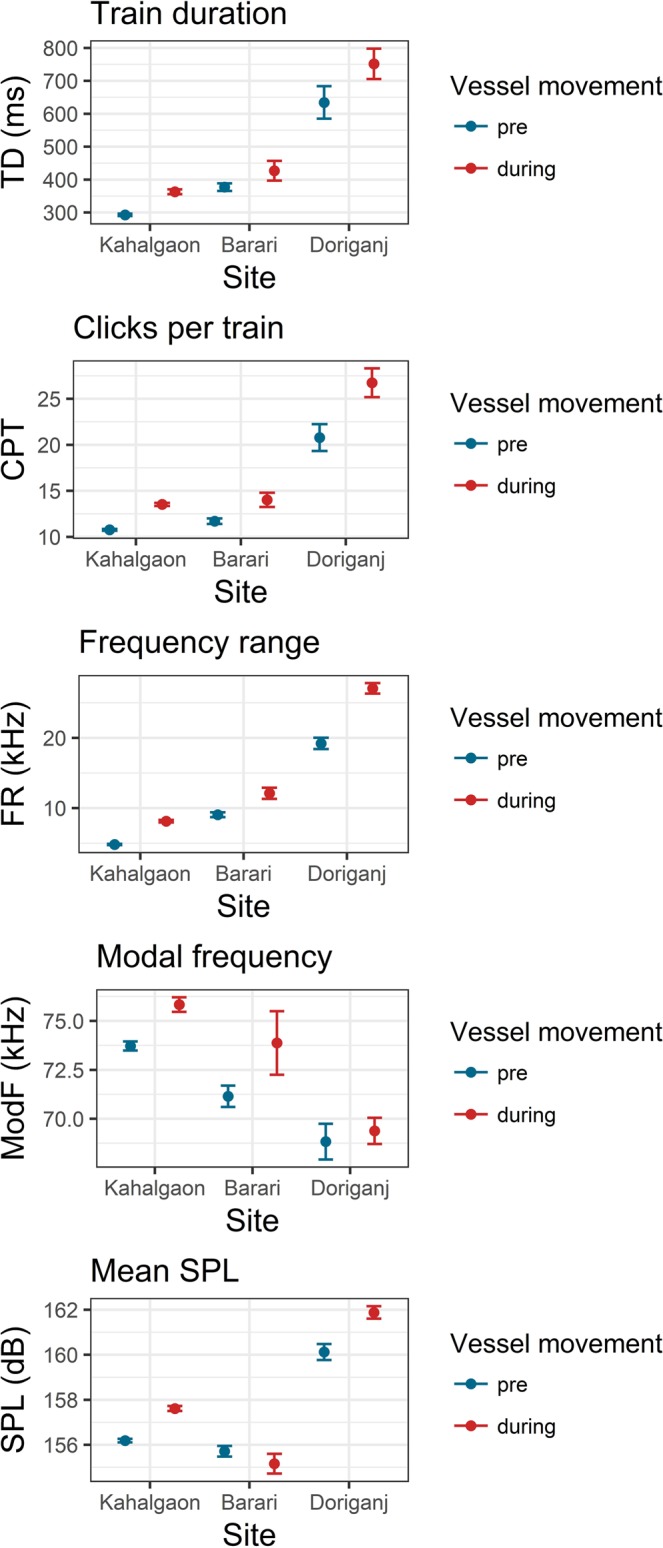
Acoustic responses with 95% CI of river dolphins to ambient noise from passing vessels across three sites: Kahalgaon (deep-noisy), Barari (shallow-quiet), and Doriganj (shallow-noisy). Elevated responses to higher levels of noise were seen for all acoustic properties except for modal frequency, which dolphins lowered in noisier conditions. All differences were statistically significant (supporting information S6).

River discharge conditions in the peak dry-season ranged over an order of magnitude: from 470 m^3^/s (shallow-noisy site) to 6430 m^3^/s (deep-noisy site). Maximum river thalweg depths were 7 m and 35 m at these sites. Average (±SE) vessel noise levels also varied, from 101.54 (±0.01) dB re 1 μPa in the ‘shallow-noisy’ site to 110.61 (±0.01) dB re 1 μPa at the ‘deep-noisy’ site (March 2018). At the ‘deep-noisy’ site, the noise levels increased from about 96 dB re 1 μPa to 110 dB re 1 μPa from November 2017-March 2018. Differences in acoustic responses between the ‘pre’ and ‘during’ phases were significant and varied with river depth and noise levels across sites (statistical results in Table S4 in Supplementary material S6). Relative changes in TD, CPT, and FR were twice higher, PF baseline and response was 6–8% lower, and SPL was 3–5% higher at the ‘shallow-noisy’ site than the ‘deep-noisy’ site. Differences were much higher at the ‘shallow-noisy’ site than at the ‘deep-noisy’ and ‘shallow-quiet’ sites (Fig. 4). Pre-vessel baseline acoustic responses of dolphins also differed significantly across the three sites (Table S5 in Supplementary materials S6).

### Masking range of dolphins

**Figure 5:**
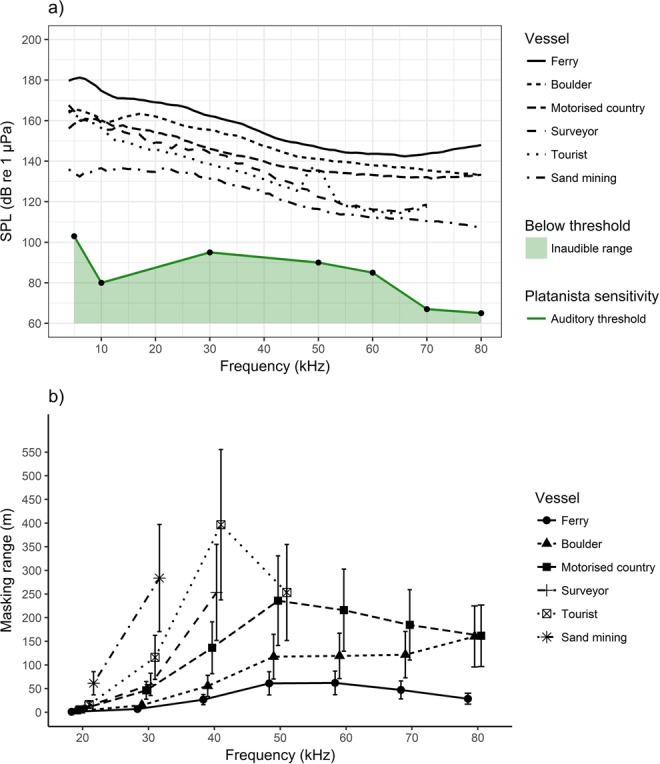
(**a**) The graph depicts the sound source level of different types of vessels at 1 kHz frequency bins, with line in green indicating the threshold level of hearing for *Platanista*^59^. The green shaded area represents the inaudible zone. (**b**) Maximum masking ranges varied between 50 m and 400 m for frequencies from 40 to 80 kHz. Error bars represent 95% CI and are based on the “transmitter” dolphin’s distance (modelled for 2–10 m) from the vessel. The strongest masking effects on *Platanista* clicks were estimated for slow-moving vessels such as ferries and motorized country boats.

Modelled sound source levels of cavitation noise decreased as frequencies increased from 4 kHz to 80 kHz, with reference to the hearing sensitivity of *Platanista* (Fig. 5a). Cavitation noises from all vessels were well above the auditory threshold of the river dolphin. This meant that all motorised vessels we measured (which included every vessel type moving in the river) were found to produce at least some frequencies in the range known to be audible to *Platanista* dolphins. Medium-sized, slower-moving ferries, boulder-carrying barges, and motorized country boats had the highest SPL whereas large tourist vessels and hydrographic surveyor vessels had lower SPLs based on our model (Fig. 5a). The difference between the auditory threshold levels of Ganges river dolphins (inferred from 59) and the SPL of vessels varied with a mean difference of 43 dB for 50 kHz, 41.6 dB for 60 kHz, 58.5 dB for 70 kHz and 65.5 dB for 80 kHz. Therefore, regardless of the type of vessel plying on the river, dolphins could certainly perceive all passing vessels from a sufficient distance, especially in the region of their highest hearing sensitivity (50–80 kHz).

The masking range of echolocation clicks of dolphins corresponded closely with the sound pressure levels of vessels. Ferryboats and boulder barges with uncased engines and poorly designed propellers had the strongest masking effect on *Platanista* clicks (Fig. 5b). Vessels with more efficient propeller designs like tourist vessels or hydrographic survey vessels had a moderate effect on the masking range at lower frequencies (<50 kHz) (Fig. 5b), but interfered with river dolphin clicks due to their use of SONAR frequencies at 50 kHz (Fig. 5b). Mean masking range at frequencies beyond 40 kHz varied from 50 m to 400 m, being conditional on engine speed and propeller design.

### Metabolic costs of altered acoustic activity

**Figure 6:**
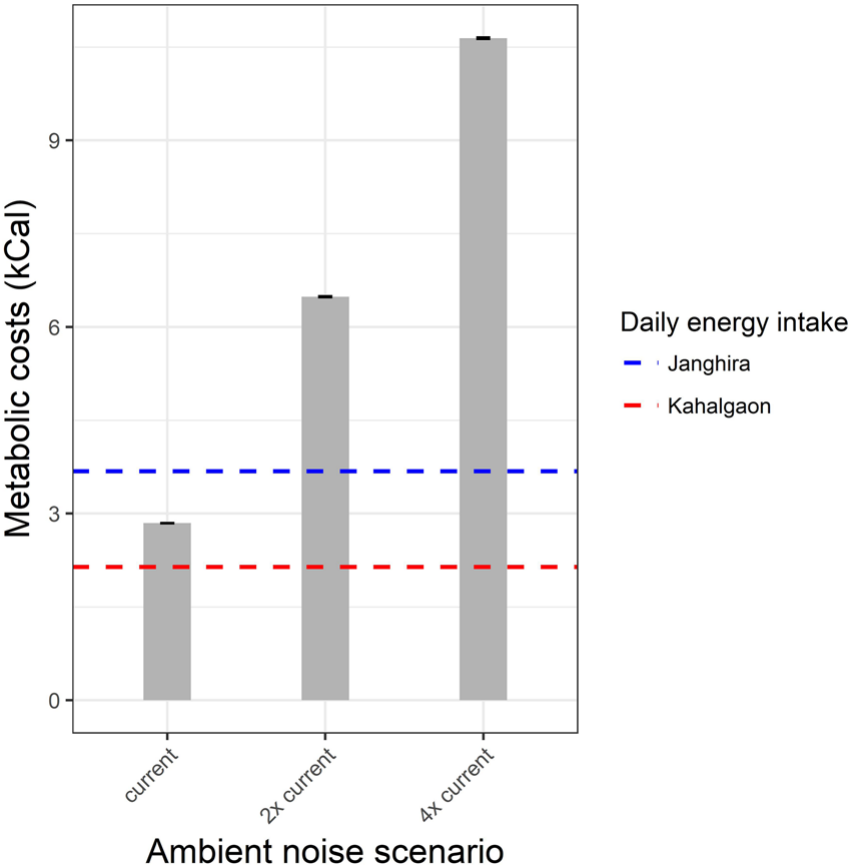
A comparison of the metabolic costs of clicking in altered acoustic environments between Kahalgaon (deep–noisy) and Janghira (deep–quiet). In Kahalgaon, where feeding rates were lower than Janghira, maintaining continuous altered acoustic activity in current ambient noise levels was costlier, given the natural feeding intake of a dolphin (dashed lines).

Overall levels of “altered acoustic response” indicated increased acoustic activity from 1.06 to 3.74 times when vessel noise levels would have doubled or quadrupled from current noise levels (Fig. 6). At the ‘deep-quiet’ and the ‘deep-noisy’ site, energy intake rates from dolphin feeding events were estimated at 3.68 kCal and 2.14 kCal per day, respectively (Fig. 6). These estimates indicated a nearly 40% less energy intake (deficit) in noisy conditions than quiet conditions, when depth was not limiting. Estimated mean energy expenditure for 12 hours of continuous acoustic activity (based on observed click-rates) at current noise levels was 2.845 ($\pm$SE 0.005) kCal, which would translate into an energy deficit of 20% in the ‘deep-noisy’ site but not exceed current energy intake by dolphins in the ‘deep-quiet’ site. In the event of doubling of ambient vessel noise, we estimated mean metabolic costs of altered acoustic activity at 6.47 kCal $(\pm$SE 0.011). This would mean a 76% higher metabolic cost than baseline energy intake in the ‘deep-quiet’ and 200% higher cost in the ‘deep-noisy’ sites. For a quadrupling of noise levels, the metabolic costs (estimated at 10.63 kCal ($\pm$SE 0.013)) would exceed the baseline energy intake by 190% in the ‘deep-quiet’ and by 400% in the ‘deep-noisy’ sites. Our estimates indicate biologically severe increases in metabolic costs under potential scenarios of exponential increases in noise levels.

## Discussion

### Impacts of underwater noise and reducing water depth

Our study established that increase in underwater noise due to motorised vessels resulted in major alterations to acoustic responses, strong masking of echolocation clicks, and high metabolic costs to river dolphins in the Ganga River. Vessel noise impacts were the strongest at low water depth in the dry-season. Significant increases in vessel traffic during the dry-season (March) indicate that dolphins might suffer from the combined impacts of high underwater noise and declining river discharge, and corresponding declines in fish prey availability due to competition with fishing activity (Fig. S2 in Supplementary material S2). Our findings were validated by evidence from time-series data for a reference site and spatially explicit comparisons across three sites spanning a gradient of ambient noise levels and depths.

When vessel traffic and ambient noise levels were low (<4–5 vessels per hour), *Platanista* compensated for masking of clicks and lost echo-perception by enhancing acoustic activity. However, when vessel traffic and noise levels increased above this limit, *Platanista* suppressed their acoustic activity and did not significantly alter their clicks. This response could be interpreted as a point where metabolic costs of enhancing or altering their acoustic activity and click properties were far greater than any benefits, as ambient noise remained high during frequent or almost continuous vessel passage. Continuous noise exposure would compel dolphins to substantially alter baseline acoustic activity, leading to significant opportunity costs of foraging and other social behaviours.

Dolphin train durations and click rates showed a large increase from baseline levels, but peak frequency and sound pressure level showed limited change. An 8% change from baseline levels may not appear significant, but frequency and SPL are biologically constrained acoustic properties, and even minor shifts may result in disproportionately high metabolic costs, than enhanced click-rates^60^. Dolphins emitted clicks at lower frequency (PF) and higher SPL in the presence of vessels when river depth was higher. But at lower depths, dolphins showed an inverted response to vessel noise: PF increased and SPL decreased at constant noise exposure. Increasing PF would allow dolphins to resolve finer details in close proximity during noise exposure. In shallow waters, low frequency sounds (longer wavelengths) are reflected at the water surface and also absorbed by the substrate. However, high frequency sounds, by virtue of their small wavelengths, are able to propagate in water without risk of interference by the water surface or substrate. Therefore, in shallow environments with high clutter, an animal’s echolocation clicks would generally be emitted at higher frequencies. However, a simultaneous increase in SPL to compensate for masking when PF is high would lead to unwanted and deleterious impacts on river dolphins. Since increasing SPL would mean increasing the amplitude of the sound wave, high frequency sounds have a higher probability of colliding with suspended sediments and high clutter in a shallow water environment. These collisions would lead to greater backscatter/reverberation, thus limiting the utility of high frequency clicks in shallower environments^5,41,52,61^.

Cavitation noise frequencies produced by propellers of different vessels overlapped completely with the broadband clicks of *Platanista*. In addition, the large difference observed between the SPL of vessels and auditory threshold level of *Platanista* indicates that most vessels moving on the river would be perceived as “very loud” by the dolphins, especially in the auditory region of its highest sensitivity (50–80 kHz). However, masking ranges for different vessels were related to sound source levels of cavitation noise, at particular frequencies of sound. Our model for masking range predicted that when a “loud” vessel, e.g. a ferry, would pass a “transmitter” dolphin, a “receiver” dolphin would have to be within 5, 25, and 50m from the transmitter in order to hear its 20, 50, and 70 kHz click frequencies. In future scenarios of cumulative increases in underwater noise, masking ranges could considerably increase and hinder communication between river dolphins, especially for mating pairs or mother-calf interactions^62,63^. Based on the size of prey and energy flux density of a dolphin’s echolocation click, our model predicted prey detection distances to be between 20 and 140 m. In this range, prey detection by dolphins will also be negatively affected by masking from increased noise.

We modelled metabolic costs of *Platanista* with conservative energy loss assumptions. As *Platanista* are continuous click emitters^51,64^ and live in shallow habitats, metabolic costs of clicks were expected to be lower than for other dolphin species of similar size^35,65^. Despite this assumption, and not modelling movement-related costs, our models consistently estimated costs of enhanced acoustic activity under increased noise levels to be far greater than the average daily energy intake of *Platanista*. Our model predicts that *Platanista* would need to feed on 2–4 times more prey (8–16% of body weight) per day to compensate for energy loss in responding to doubling or quadrupling of daily underwater noise levels. In reality, such increases in prey intake are not possible due to satiation, as dolphins cannot eat more than 4% of their body weight in one day^66^. As a result, deficits in energy reserves may be seen in *Platanista* in the dry-season: at low water depths and high vessel traffic. Intensive fishing effort and overlap with dolphins also increases during the dry-season^67^. At high underwater noise, overlap with fishing activity could restrict prey capture ability, elevate stress, alter foraging behaviour, and increase bycatch in entangling fishing gears. Disorientation or fatigue from prolonged response to underwater noise is also likely and could result in higher probability of propeller hits. Mortality of dolphins from propeller hits is not uncommon, for example, in the Hooghly River (West Bengal, India)^68^, where boat traffic is much higher than our study area. The impacts of such indirect stressors in addition to frequent underwater noise could intensify in drought years with poor river depths. Exposure to noise could also aggravate the high background mortality rates of Ganges river dolphins (especially calves) in the peak dry-season. These cumulative stresses may be detrimental to the wellbeing of river dolphins in the Ganga River waterway.

### Combined impacts and potential mitigation measures

Naturally deeper river channels might buffer underwater noise impacts, and this effect of depth must be understood in the right context. For waterways, maintenance dredging is conducted in the dry-season to deepen channels, but that may not mitigate underwater noise impacts. On the contrary, dredging could contribute to stress in river dolphins by physical and biogeochemically disturbing riverbed sediment and impacting fish prey^43^, even though dredging-related underwater noise levels are relatively low frequency and could be localised^14,43^. Our field observations indicate about three-fold increases in dive-times (indicating stress) and avoidance of dredged channels by dolphins. In this regard, the efficacy of mitigation measures such as dredge-curtains in reducing noise or sediment disturbance to dolphins needs to be experimentally tested in future studies.

River dolphins in their natural habitats need a combination of deep and shallow river segments (for resting, feeding, and other activities)^67,69^. Dredging and underwater noise can affect dolphin habitats both physically and acoustically. Hence the focus should be on maintenance of natural river channel depth profiles by providing ‘ecological flow regimes’ to sustain river dolphin populations. This involves maintaining adequate flows into the river from upstream irrigation dams and barrages on the Ganga river main stem as well as inflows from tributaries, which would depend on water demand management across sectors. Our study thus expands the concept of ‘ecological flow regimes’ to include prescriptions on limiting anthropogenic underwater noise impacts on freshwater fauna. To our knowledge, this is the first combined investigation of underwater noise impacts on metabolic costs to dolphins, in relation with river depth.

Our study provides a scientific basis to identify how noise impacts could be minimized with a precautionary conservation approaches and mitigation measures. These range from reducing waterways intensification (e.g. no dredging, downscaling vessel traffic to limit underwater noise) to technological improvements (improving propeller efficiency to cut down cavitation noise). Technological improvements may not only help reduce production of cavitation noise, but also improve fuel efficiency for vessels^70^. Yet, only curbing noise at-source may not be enough to avert overall impacts of vessel traffic. For example, despite lower cavitation noise, larger industry-made vessels need more draft (i.e. more dredging), can cause greater risk of physical injury, and expose river dolphins to constant active SONAR. Assessing trade-offs between efficiency, vessel capacity, and technological improvements on the one hand, and background maintenance costs on the other (e.g. from dredging), is thus essential to reduce and mitigate risks to *Platanista* from vessel traffic.

Our study contributes rigorous scientific knowledge to ongoing debates on the ecological impacts of large-scale waterways development in regulated rivers in the Indian subcontinent. The endangered Ganges river dolphin is a flagship species for river conservation and also India’s “National Aquatic Animal”. Forthcoming species recovery and conservation action plans for the species need to recognize underwater noise as an important and emerging threat, and work towards scientific monitoring and mitigation of noise impacts on endangered riverine fauna.

## Materials and Methods

### Study area

We conducted field studies at four sites along the Ganges River in Bihar, India from November 2017 to April 2018. All sites lie along India’s National Waterway No. 1 (NW-1)^54,55^. These sites hold among the highest known densities of Ganges river dolphins (2.5–3.0 dolphins/km) in their range^46,67^. We chose our reference site as Kahalgaon (27.26N, 87.236E) in the Bhagalpur district of Bihar, where we monitored dolphin responses, vessel traffic, ambient noise levels, river discharge, depth, and fishing intensity throughout the study period. Kahalgaon is one of the deepest stretches in the Ganga River (maximum mid-channel depths at 35–40 m in the dry-season), and experiences regular ferry movements. The other sites chosen for comparative studies included Barari and Janghira in the Bhagalpur district and Doriganj in Chapra district of Bihar (Supplementary material S1: Fig. S1). A clear gradient of vessel traffic, ambient noise levels, and river depth was seen across these four sites. Accordingly, we classified them as: Kahalgaon (deep: 35 m, noisy); Janghira (deep: 10–15 m, quiet); Barari (shallow: <4 m, quiet), and Doriganj (shallow: <7m, noisy). Sites below a median ambient noise of 90 dB re 1 μPa were classified as “quiet”, and above this threshold, sites were classified as “noisy”. This criterion was chosen at 20 dB above the known baseline auditory threshold of *Platanista*^59^, at which we expected behavioural responses^71^. We classified Janghira and Barari as “quiet” sites because vessel passage was zero in Janghira and the frequency of vessel passage at Barari was extremely low during our recording periods. The river stretch (presently 70 km long) from Sultanganj to Kahalgaon in the Bhagalpur district was designated as the Vikramshila Gangetic Dolphin Sanctuary (VGDS) in 1991^72^, in which two of our study sites, Kahalgaon and Barari, were situated. Details of the study sites are provided in Table 1.

**Table 1:** Sampling details at sites where acoustic and behavioural responses of Ganges river dolphins were assessed through sound recordings and field observations. Sites with less than 7m of water were considered as ‘shallow’, and sites with more than 10m depth were called deep site.

Site characteristics	Ambient noise level (dB re 1 μPa) Mean (±SE)	Site Name (sampling effort, in hours)	Period of field studies	River depth (min-max, m)	Vessel traffic (average no. of boats per hour)	Other observations & remarks
Deep, Noisy	96.43 (0.01) to 110.61 (0.01) from November to March	Kahalgaon (n = 489)	November 2017 to March 2018	2–33	4	Noise recordings of different vessel types, fishing activity, change in river depth/discharge
Shallow, Quiet	86.9 (0.04)	Barari (n = 67)	November 2017 to March 2018	1–5	1	—
Deep, Quiet	40.58 (0.04)	Janghira (n = 96)	February 2018	1–19	0	—
Shallow, Noisy	101.95 (0.01)	Doriganj (n = 9)	March 2018	1–7	11	—

### River discharge and boat traffic levels

For each month of sampling across different sites, river depths and channel width were measured by doing cross-sectional measurements of the river channel using a hand-held depth sounder (HONDEX PS 7) and GPS unit (GARMIN *e-trex* 30). Manning’s equation for open channels was used for river discharge estimation from cross-section depth data, riverbed slope, and an alluvial roughness coefficient^73,74^. Detailed information on river discharge estimation is provided in Supplementary material S2. Changes in river discharge and depth were estimated through the dry-season from November 2017 to March 2018 for our reference site (Kahalgaon).

### Recording ambient noise levels from vessel-induced underwater noise

We used a hydrophoneAQH-200K (Aquasound Inc., Kobe, Japan; sensitivity: −220 dB re 1 V/μPa; frequency response: 20 Hz–200 kHz), paired with preamplifiers (Aquafeeler III & RESON VP2000) and portable PCM recorders (KORG MR-2 & TASCAM DR-100 mk III), to record and characterise ambient noise levels at the four study sites and vessel sound source level. We also visually recorded the passage time of each vessel from 06:00 AM to 06:00 PM on each recording day, to estimate the relationship between vessel traffic and ambient noise level. Our recordings covered a total of 125 individual boat passage events, with each event lasting 2–5 minutes. Details of conditions for ambient noise levels and recording setup are given in Supplementary material S3.

### Dolphin acoustic responses to ambient noise

Specialized passive acoustic loggers known as CPODs (Cetacean and POrpoise Detection devices, www.chelonia.org) were used to log the clicks produced by Ganges River dolphins. CPODs have been a standard PAM technique for several recent cetacean studies (see Supplementary material S3). Acoustic responses of dolphins can be measured from CPOD log files and the following variables extracted: train duration (TD), clicks per train (CPT), frequency range (FR), modal (peak) frequency (PF), and average sound pressure level (SPL). Table S1 in Supplementary material S4 provides the definitions of acoustic responses that we analysed. All acoustic response data was categorised into “pre-vessel passage” and “during-vessel passage” phases from direct visual observations. The ‘pre’ phase indicated periods before any vessel approached dolphins in the recording area (which also included periods with no vessel movement). The ‘during’ phase was the actual ‘response’ phase indicating periods from the closest point of approach to when vessels completely passed the dolphins in the recording area. In the ‘post-vessel passage’ phase, altered acoustic responses remained similar to the ‘during-passage’ stage for lag periods of 1–2 minutes, until *Platanista* returned to baseline acoustic activity states. The time taken to return to ‘pre’ vessel periods of acoustic response was dependent on the intensity of vessel movement in the river, with higher vessel movements increasing the lag time. Hence we focused our analysis on the ‘during’ phase as it could be defined well, based on the points of approach and departure from dolphin locations. Pre- and during-phase data for vessel passage events were paired to compare baseline acoustic activity (pre) and acoustic responses (during) relative to the baseline. We checked how acoustic responses of river dolphins changed from the ‘pre’ to the ‘during’ phase for all vessel passage events. Total CPOD recordings covered 489 hours over 4 months. Time series data on these variables were graphically analysed in relation to vessel traffic (boats per hour) for each recording day, for our reference site (Kahalgaon). Changes over time were also interpreted in relation to changing river discharge and fishing intensity with the progress of the dry-season.

We used non-parametric tests (Mann-Whitney U-test, Cliff’s Delta estimates of “effect size” of responses) to determine statistical significance of differences in acoustic responses from the pre- to the during-phase (Supplementary material S5). Our choice of non-parametric tests was due to the inevitable measurement errors involved in field-based recording of acoustic responses. The measurement errors meant that the relative ordinal differences in values of acoustic response variables were the right choice for analysis than the actual values. We also used non-parametric measures of effect size to assess the magnitude of relative responses, over showing only the statistical significance of differences.

Hydrophone recordings of peak frequencies have the highest accuracy when all recorded clicks are ‘on-axis’, i.e., a dolphin is directly facing the hydrophone. However, this condition is impossible to ascertain in the natural habitat (recording context) of river dolphins. *Platanista* echolocation clicks also show high directionality, which would affect the sampling coverage and measurement precision of the CPOD and hydrophone recordings. Acoustic response data obtained from the CPOD would thus involve complex measurement errors arising from variable effects of unmeasured covariates on the response variable, which are difficult to estimate. This variability stifles the ability of the mean response (50^th^ quantile, used in ordinary regression models) to capture the influence of the measured covariate. We therefore used quantile regression models to statistically estimate regression relationships at higher quantiles of the response variable to the measured covariate(s) of interest^75^. Quantile regression helped us estimate the effects of proportional changes in ambient noise level on different levels of altered acoustic responses of river dolphins. Acoustic variables (responses) and ambient noise level (predictor) were rescaled to indicate proportional change in acoustic response relative to baseline click variables as {(‘*during’ − ‘pre’*)/‘*pre’*} (n = 125). Quantiles in the 50^th^ to 90^th^ quantile range were analysed for all responses, except for PF, where lower quantiles (within the 10^th^ and 40^th^ quantiles) were analysed in relation to ambient noise as well. All analyses were conducted in the software R version 3.4.2^76^.

### Effects of river depth on acoustic responses to ambient noise: multi-site data

Baseline levels of river dolphin click properties might vary across sites due to localized acoustic adaptation to natural and anthropogenic background noise sources. So, we tested for statistical differences across sites for all acoustic response variables between baseline levels (in quiet periods), and altered acoustic responses during noise exposure from vessel passage. For this we used graphical comparisons and Kruskal-Wallis tests (a type of non-parametric ANOVA)^77^. Kruskal-Wallis test results were qualified with non-parametric Bonferroni–Dunn tests (or Dunn tests)^78^. These tests helped estimate the degree of difference between acoustic responses across ‘shallow-noisy’, ‘shallow-quiet’, and ‘deep-noisy’ sites, relative to their site-specific baseline click properties, ambient noise levels, and river depth/discharge conditions. We could not compare data from ‘deep-quiet’ sites because of limited sample sizes (events of vessel passage). Details of the statistical comparisons are provided in Supplementary material S6.

### Vessel source level and masking range

Masking range is defined as the distance beyond which acoustic signals, i.e. high frequency clicks emitted by “transmitter” dolphins, would become inaudible to other “receiver” dolphins, in an environment where they are exposed to anthropogenic noise^62,79^. For estimation of masking range, sound source levels for different vessels were calculated using the shallow water propagation model developed by^80^ to account for higher reverberation and sound absorption^4,61^. We estimated the sound source level from the cavitation noise produced by six types of vessels that regularly moved on the river. Environmental variables such as pH, temperature, salinity, depth, and distance of sound source were included in the model (model equations and details of estimation are provided in Supplementary material S7). The model included the assumption that the transmitter dolphin was within 10 m of a particular type of vessel. We assumed 10 m only as a reference distance for modelling the masking range. This choice was to avoid estimation errors at larger distances and to still represent field observations in which dolphins were often observed in very close proximity to passing vessels. Known root-mean-square (rms) sound source levels of Ganges dolphin clicks (160–170 dB re 1 μPa at 1 m) at a peak frequency of 74 kHz were used, based on^50^’s estimate for the same study area. However, for masking range estimation, sound source levels of dolphin clicks were needed at specific frequency intervals from 20 kHz to 80 kHz (frequencies corresponding with ambient noise). For this we first isolated a single ‘on-axis’ echolocation click and used the source level of the dolphin at 74 kHz as the reference value to calculate the approximate distance of the dolphin from the recording point (Fig. S3 in Supplementary material S7). With this reference, dolphin sound source levels were calculated from 20 to 80 kHz, in frequency bins of 10 kHz.

### Estimation of metabolic costs of dolphins in noisy environments

There are no data on the metabolic costs of producing echolocation click trains for Ganges river dolphins. So, we used information on metabolic costs of clicking in bottlenose dolphins as a crude baseline^81^. Assuming similar metabolic costs, we estimated the cost of producing each individual click for a dolphin. From the CPOD data we were able to distinguish feeding events from normal clicking activity^82^. Feeding rates of Ganges river dolphins over 24-hour continuous recording periods were calculated for the ‘deep-noisy’ and ‘deep-quiet’ sites (Kahalgaon and Janghira). Based on the estimated detection range of the CPOD hydrophone, and the number of dolphins present around the CPOD location, we estimated the potential number of feeding events per dolphin per day. This was corrected to a 400 m distance of river that we assumed a dolphin to traverse on one day, from our visual and acoustic observations. Ganges river dolphins feed on small fishes with average lengths of 6 cm^50^. We estimated the caloric content in a fish of that size^83^, and multiplied it by estimated daily feeding rates to assess the total energy intake (in calories) of an individual dolphin at the two sites.

To model energetic requirements of Ganges river dolphins in noisy conditions, we estimated total metabolic costs of altered acoustic activity in response to ambient noise levels. For this we used the energetic costs of producing a single click derived from bottlenose dolphins^26^. We then used a regression tree analysis^84^ to estimate thresholds of modified acoustic responses to changes in ambient noise levels (Fig. S5 in Supplementary material S7). From the outputs of the most parsimonious tree models, changes in acoustic responses to ambient noise levels under three scenarios were identified: (1) current noise level; (2) doubling; and (3) quadrupling of the current noise level (Supplementary material S7). We modelled the metabolic costs for a 12-hour time period, which coincided with the daily vessel movement in the river. Changes estimated for the 5 simultaneous acoustic responses were added to estimate a net “altered acoustic response” under the three scenarios, by adapting the method of^26^.

## Supplementary information

Supplementary Information

## Data Availability

Data is available upon reasonable request to the corresponding author.
