# Supplementary material for: Interacting effects of vessel noise and shallow river depth elevate metabolic stress in Ganges river dolphins
Source: Sci Rep. 2019 Oct 28;9:15426. doi: 10.1038/s41598-019-51664-1 (PMC6817857; doi:10.1038/s41598-019-51664-1)
Supplement: Supplementary file 1 — Supplementary Information [file 41598_2019_51664_MOESM1_ESM.docx]

**Interacting effects of vessel noise and shallow river depth elevate metabolic stress in Ganges river dolphins**

Mayukh Dey^1^*, Jagdish Krishnaswamy^2^, Tadamichi Morisaka^3^, Nachiket Kelkar^2^

^1^ P.G. Program in Wildlife Biology and Conservation, National Centre for Biological Sciences-TIFR, GKVK Campus, Bellary Road, Bangalore 560065, Karnataka, India. Email: [mayukh.d27@gmail.com](mailto:mayukh.d27@gmail.com)*Corresponding Author.

^2^ Ashoka Trust for Research in Ecology and the Environment (ATREE), Royal Enclave Srirampura, Jakkur PO, Bangalore 560064, Karnataka, India.Email:
[jagdish@atree.org](mailto:jagdish@atree.org), [nachiket.kelkar@atree.org](mailto:nachiket.kelkar@atree.org)

^3^ Mie University, 1577 Kurimamachiya-cho, Mie 514-8507, Japan. Email: [chaka@bio.mie-u.ac.jp](mailto:chaka@bio.mie-u.ac.jp)

**Keywords:** Ganges river dolphins, underwater noise, vessel traffic, shallow rivers, waterways, acoustic behaviour, masking range, metabolic costs, ecological flows

Supplementary Material S1.

Figure S1. Locations of the study sites: Bhagalpur, Kahalgaon, and Janghira in the Bhagalpur district, and Doriganj in the Chapra district, along the Ganga River in Bihar.


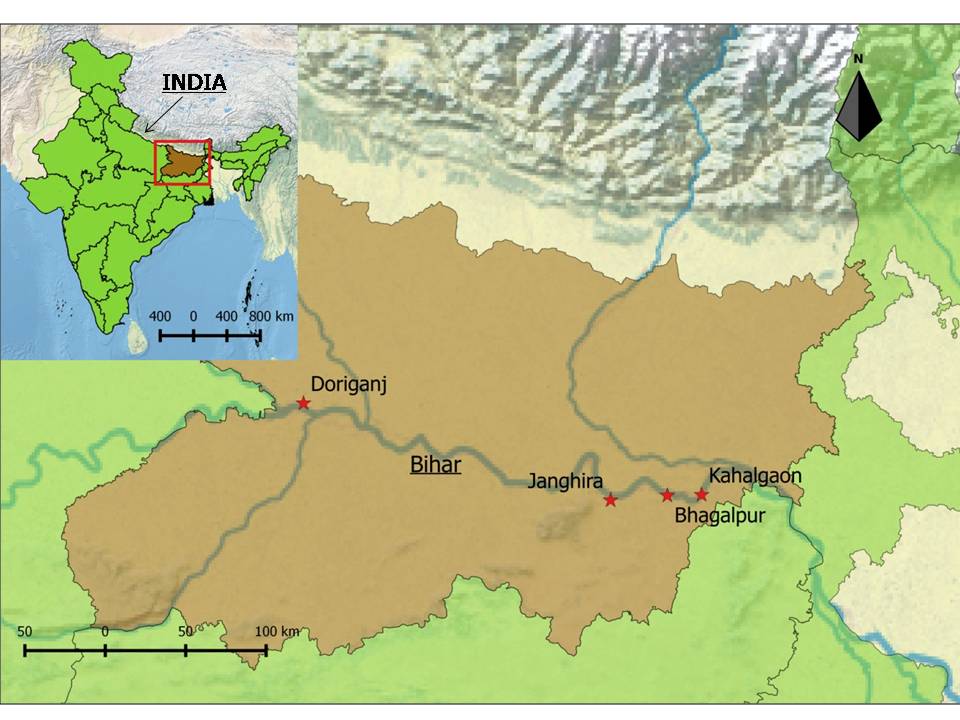


**References**

1. Government of India. National Waterway (Allahabad-Haldia Stretch of the Ganga-Bhagirathi-Hooghly River) Act (No. 49 of 1982). October 18, 1982 (1982).
2. Government of India. National Waterways Act (No. 17 of 2016). Registered No. Dl—(N) 04/0007/2003—16. March 26, 2016, 15 p (2016).
3. QGIS Development Team. QGIS Geographic Information System. Open Source Geospatial Foundation Project. <http://qgis.osgeo.org> (2018)

**Supplementary Material S2.**

**Details of discharge estimation and fisheries data.**

*River depth, channel width, and discharge data*

For each month of sampling across different sites, river depths and channel width were measured by doing cross-sectional measurements of the river channel using a hand-held depth sounder (HONDEX PS 7) and GPS unit (GARMIN *e-trex* 30). All cross-sectional depth measurements were conducted at 10 m intervals from the left bank to the right bank of the river. For discharge estimation, we followed classical procedures by employing the Manning’s equation for open channel discharge in large rivers, based on (1; 2; 3)’s methods. River discharge (volumetric flow rate, in m^3^/s) was calculated from river bed-slope, *S* assumed equal to 6 cm/km and the alluvial roughness coefficient *n* equal to 0.035. These two constants are needed for estimation of Manning’s discharge, and were obtained from (4)’s geomorphological study of the Ganga River and from standardized tables provided in (3) respectively. Manning’s discharge was calculated from the equation:

$$Q= \frac{A}{n}*R^{2/3}*S^{1/2}$$

where, $Q$ is the total river discharge, A the cross-sectional area, R the hydraulic radius, calculated as the quotient of cross-sectional area divided by the wetted perimeter, and S the river bed slope. Changes in discharge and cross-sectional characteristics of the river were estimated from November 2017 to March 2018 for Kahalgaon, to represent the decline in river flow with the progress of the dry season.

*Fish catch data at Kahalgaon during the study period*

Fish catch data were obtained from logbook-based monitoring surveys conducted at the Kahalgaon fish market during the study period (Oct 2017 to March 2018; Kelkar et al., unpublished). The total yield from this period was found to be proportional to fishing effort (Kelkar, N., pers. comm.). Hence, catch-per-unit-effort could be assumed to be a crude, but consistent index for prey availability. Catch per unit effort (CPUE; kg of fish per fisherman per day) was calculated for species whose sizes are within the known average prey size limits of Ganges dolphins (reported by 5; 6), and month-to-month variations in CPUE were estimated. River hydrology and fisheries catch data were used to assess the interaction of noise impacts with changing habitat and prey availability for dolphins in their dynamic river habitat.

*Changes in fish prey availability*
 We assumed that fish catch per unit effort was proportional to relative abundance of fish prey (fish species <20 cm at maturity) at Kahalgaon. Seasonal data showed that after late February, there was a drastic decline in fish catch per effort (Figure S2), corresponding with decline in dry-season river discharge. At this time, when there was a substantial increase in vessel traffic and noise levels in the Ganga River, prey availability also reduced.

**Figure S2.** Fish catch per unit effort (kg per fisher per day) at Kahalgaon during the study period. Fish catch per unit effort was only calculated for species whose sizes make them potential dolphin prey. Note the drastic reduction in catch per unit effort from late February onwards, after which noise levels increased and river depth declined as well.


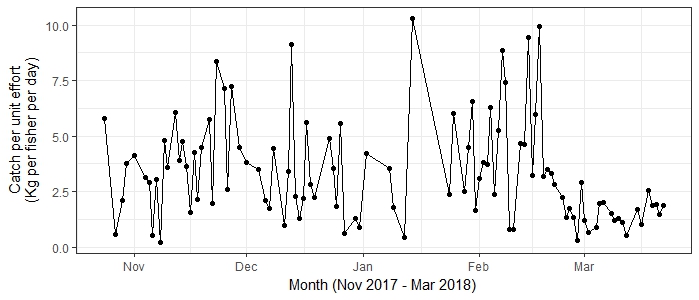


**References**

1. Chow, V.T. Open-channel hydraulics: New York, McGraw-Hill, 680 p (1959).
2. Williams, G. P. Bank-full discharge of rivers. *Water Resources Research*, *14*(6), 1141-1154 (1978).
3. Arcement, G. J., & Schneider, V. R. Guide for selecting Manning's roughness coefficients for natural channels and flood plains. United States Geological Survey Report No. FHWA-TS-84-204, for the Federal Highway Administration, USA, p. 72 (1984).
4. Singh, M., Singh, I. B., & Müller, G. Sediment characteristics and transportation dynamics of the Ganga River. *Geomorphology*, *86* (1-2), 144-175 (2007).
5. Kelkar, N., Krishnaswamy, J., Choudhary, S. & Sutaria, D. Coexistence of fisheries with river dolphin conservation. *Conservation Biology*, 24, 1130–1140 (2010).
6. Kelkar, N., Dey, S., Deshpande, K., Choudhary, S.K., Dey, S., & Morisaka, T. Foraging and feeding ecology of Platanista gangetica: an integrative review. Mammal Review, 48, 194-208 (2018).

**Supplementary Material S3.**

*Details of CPOD deployment and recordings*

At each site, two CPODs were simultaneously deployed using 10 kg weights suspended from small fishing boats (non-motorised) that served as mooring stations for a period of 8-24 hours per survey replicate (day), resulting in a total of 689 hours of recordings conducted over 4 months across four sites. The optimum depth for deploying CPODs was chosen at 0.4 times the depth of the water column. This depth was chosen based on (1) who indicated that river dolphins emit the maximum acoustic clicks in that section of the water column. The two CPODs were deployed at locations about 500 m apart, based on a 250 m assumed radius of device coverage, which was corroborated by data on sound pressure levels of Ganges river dolphins logged in the CPOD data files. The sound source levels dolphins were estimated from Kahalgaon and Bhagalpur during an earlier study using A-Tag device by Morisaka et al. (unpublished) and (1) which served as reference values to calibrate CPOD outputs.

Further, the estimated accuracy of CPOD recordings of dolphin clicks would be conditional on the assumption that the river dolphins were facing the device when they were clicking (i.e. on-axis clicks were recorded). As this could not be assumed as a constant condition, care was taken to select calls extracted from the CPOD software (<http://www.chelonia.co.uk/>), by comparing estimated values with known reference ranges of acoustic variables from earlier studies here by Morisaka et al. (unpublished).

The CPOD software classified all sound sources in the CPOD file as click trains coming from ‘NBHF’ (narrow-band high frequency) or ‘other cet’ (other cetaceans) and clicks trains from unknown sources, as ‘unclassified’. The software further classified the quality of each train type into ‘high’, ‘medium’ or ‘low’ (2). After data file extraction, click trains belonging to all classification types and quality levels were visually sorted (from graphical displays in the CPOD software) for removing false positives (i.e. sounds from unknown or ambiguous sources being classified as ‘other cet’), which were reported by (3). Variables estimated for click trains that were chosen as response variables (i.e. dolphin responses to ambient underwater noise from vessels) included modal frequency of clicks (in kilohertz: kHz, corresponding to the peak frequency), received sound pressure level or SPL (in decibels: dB), frequency range (minimum and maximum kHz in a train), clicks per train (number of clicks within a recorded train), and train duration (time length of each train in milliseconds). Table 2 describes the acoustic response variables and their ecological significance for dolphins.

To look at the temporal variation in the 5 acoustic response variables (Table 2) in ‘pre’ and ‘during’ phases, time series data on these variables was plotted date-wise in relation to boats per hour. The average vessel movement per hour for each recording day was plotted as well to graphically examine the relation between the observed acoustic response relative to baseline and the number of vessels moving in the recording area. We tested for the statistical significance of differences in median acoustic response levels (ranks) in the ‘pre’ and ‘during’ phases using the Mann-Whitney test, a non-parametric method, with the alpha value set at 0.05. As this test did not offer any direct measure of ‘effect size’, i.e. the unit change in the ‘during phase’ with respect to ‘pre (baseline) phase’, we used Cliff’s Delta (4) as a non-parametric measure of effect size.

*Hydrophone recordings: vessel noise and ambient noise levels*

Noise from vessels is comprised of sounds generated from: 1) boat engines; 2) propellers (cavitation noise or the noise of bursting bubbles generated by propeller rotations); 3) noise from devices used on vessels for hydrographic surveying (e.g. SONAR);and 4) resulting reflections or aberrations in the above categories of sounds after being transmitted. Of these, source levels of engine noise are usually low in frequency (<2 kHz), whereas cavitation noise levels and ship SONAR cover the audible frequency range in dolphins, thus directly interfering with the dolphins’ sonic capabilities (5). To record ambient noise levels from the different vessels plying on the rivers, we used a hydrophone AQH-200K (AquaSound Inc., Kobe, Japan; sensitivity: -220 dB re 1 VPa; frequency response: 20 Hz–200 kHz), paired with preamplifiers (Aquafeeler III & RESON VP2000) and portable PCM recorders (KORG MR-2 & TASCAM DR-100 mk III). Each recording lasted for 1 minute and 5 recording replicates were conducted every hour, with a sampling rate of 192 KHz and 24-bit depth. Frequencies ranging from 500 Hz to 20 kHz were used for ambient noise level analysis, because sound frequencies above 20 kHz usually are contributed by river dolphins and were logged in the CPOD data files. Further, the CPOD log files also recorded the frequency ranges of cavitation noise levels, which extended well beyond 20 kHz.

The hydrophone, preamplifiers, and recorders were calibrated and used for recording at all sites, where sampling was conducted in response to events of boat passage, covering a total of 125 individual boat passage events, with each boat passage event typically ranging from 2 to 5 minutes. The hydrophone was deployed from the same anchored boat that was used for CPOD deployments at depths ranging from 2 to 5 meters below the water surface, based on prevailing depths at the recording location. The sampling rate of the recordings was 192 kHz and the total bit-depth of the recorded files was 24-bit. The files were saved in ‘.wav’ format for further analysis in R.

We noted down the start time, duration, and end time of vessels passing, from which intensity of vessel traffic for every hour was calculated, and the type of vessel plying was noted. When each individual vessel passed the hydrophone coverage area, the distance of the vessel was noted using a rangefinder (Nikon PROSTAFF 3i laser rangefinder) along with the corresponding time in seconds displayed on the recorder. This was done in order to estimate the sound source level of different vessels plying on the river. At every hour, factors which were likely to affect the absorption coefficient or α, of all sources of sound, such as temperature, salinity and pH, was noted along with the average depth of the river, which remained unchanged for every sampling station. Salinity, pH and temperature were measured using a digital handheld probe (DANOPLUS; model number: M0199720), while river depth was measured with a handheld depth sounder (Hondex^TM^, Japan).

For extracting cavitation noise data the low cut filter on the preamplifiers was set at 4 kHz and only sounds from 4 kHz till 80 kHz were used for cavitation noise calculations. The recorded sound file was viewed on Raven pro software (version 1.4) and a 1 second sound clip was used for analysis. The 1 second noise recording was selected based on the absence of dolphin clicks in the file. The sound file was then extracted and analysed in R (version 3.4.2, R Core Team 2017) using sound analysis packages such as “tuneR” and “seewave” (6). Fast Fourier Transform (FFT) algorithms were applied using the functions in ‘seewave’ package, on the 1 second sound clips which yielded a resolution of 1Hz in the frequency domain (window length=512, time resolution=1 second, window type=Hanning) (adapted from 6; 7). Using the factors, contributing to α (absorption rate), as specified by (8), along with the variables associated with shallow water propagation model (9), distance of the boat from the recording location, and the sound pressure level received at the hydrophone, the sound pressure level at the source (vessel) was calculated. The sound source level was calculated at a 1 Hz bin and later condensed into 1 kHz bin.

**References**

1. Kelkar, N., Dey, S., Deshpande, K., Choudhary, S.K., Dey, S., & Morisaka, T. Foraging and feeding ecology of Platanista gangetica: an integrative review. Mammal Review, 48, 194-208 (2018).
2. Tregenza, N. CPOD. exe: a guide for users. *United Kingdom* (2014).
3. Robbins, J. R., Brandecker, A., Cronin, M., Jessopp, M., McAllen, R., & Culloch, R. Handling dolphin detections from C-PODs, with the development of acoustic parameters for verification and the exploration of species identification possibilities. *Bioacoustics*, 25(2), 99-110 (2016).
4. Macbeth, G., Razumiejczyk, E., & Ledesma, R. D. Cliff's Delta Calculator: A non-parametric effect size program for two groups of observations. *Universitas Psychologica*, *10*(2), 545-555 (2011).
5. Cramer, E., & Lauterborn, W. Acoustic cavitation noise spectra in *Mechanics and Physics of Bubbles in Liquids* pp. 209-214 (Springer, Dordrecht, 1982).
6. Sueur, J., Aubin, T., & Simonis, C. Seewave, a free modular tool for sound analysis and synthesis. *Bioacoustics*, 18, 213-226 (2008).
7. Morisaka, T., Shinohara, M., Nakahara, F., & Akamatsu, T. Effects of ambient noise on the whistles of Indo-Pacific bottlenose dolphin populations. *Journal of Mammalogy*, 86(3), 541-546 (2005).
8. Francois, R. E., & Garrison, G. R. Sound absorption based on ocean measurements. Part II: Boric acid contribution and equation for total absorption. *The Journal of the Acoustical Society of America*, 72(6), 1879-1890 (1982).
9. Marsh, H. W., & Schulkin, M. Shallow‐Water Transmission. *The Journal of the Acoustical Society of America*, *34*(6), 863-864 (1962).

**Supplementary Material S4.**

**Table S1.** Definitions of the acoustic variables measured in the study.

| **Variable (units)** | **Definition** | **Ecological significance** |
| --- | --- | --- |
| Train duration (seconds) | The duration a series of clicks produced by dolphins, which is used for navigation, foraging and communication, lasts for. Difference between 2 click trains is usually defined by an on-board algorithm in the CPOD. | Correlated with how long (in time) a dolphin keeps clicking for |
| Frequency range (kHz) | The difference between the minimum and maximum frequency in a given click train. |  |
| Clicks per train (counts of clicks) | The number of clicks in a click train. It is not essential for clicks per train to be positively correlated with train duration. | Rate at which dolphin produces clicks, correlated with inter-click interval. Not correlated with train duration. |
| Peak frequency (kHz) | The frequency of sound wave of a given click that contains the highest power. In the recording data, it is approximated by the modal frequency (kHz), which is the frequency repeated most in a click train. | The inverse of the wavelength, or the distance unto which an animal ‘sees’. Ganges dolphins are high-frequency echo-locators, i.e. their echolocation scans short distances in the river. |
| Inter-click interval  (ICI, ms) | The time duration between two successive clicks in a given train. | When dolphins scan distances very c close to them, the inter-click interval reduces, as they produced rapid clicks to evaluate their surroundings or detect prey. |
| Attenuation | The rate at which a sound wave loses its energy as it travels through a medium. |  |
| Sound Pressure Level (SPL) | The pressure level of a sound wave expressed in decibels. | Correlated with the perception of loudness. Higher pressure levels would lead to louder sounds, which can travel further than sounds produced at lower pressure levels. |
| Sound Source Level (SSL) | The pressure level of a sound wave produced at the point of the origin. | Similar to SPL, but refers to sound emitted at the source, i.e. the emitting dolphin, or vessel |
| Detection range (m) | The range at which an animal is able to perceive and detect a sound wave | The distance at which a dolphin can potentially perceive noise generated by vessels. |
| Masking range (m) | The range beyond which the sounds waves will be masked by a louder source of sound, in most cases, human-made sounds. Masking range calculated is for each frequency in a sound wave. | Masking range refers to the distance at which clicks produced by one dolphin might become inaudible to another dolphin in the presence of a loud vessel in the surroundings. |

**Supplementary Material S5.**

**Table S2.**The results of the Mann-Whitney tests used to determine the differences and effect sizes (Cliff’s delta) of acoustic responses between the ‘pre’ (baseline) and ‘during’ (response to vessel noise) phases for different months at the reference site (Kahalgaon). P-values considered significant when P<0.05.

| **Acoustic response (pre vs. during in different months)** | **p-value** | **Estimated mean rank difference** | **Cliff’s delta (effect size: Pre -During effect)** | **Confidence Interval (Cliff’s Delta, 95% CI)** |
| --- | --- | --- | --- | --- |
| **Train duration** |  |  |  |  |
| November  December  January  March | P<0.001  P<0.001  P<0.001  P<0.001 | -73  -41  -59  -15 | 24%  15%  21%  6% | 20%– 28%  11%– 18%  19%- 24%  3%– 8% |
| **Frequency range** |  |  |  |  |
| November  December  February  March | P<0.001  P<0.001  P<0.001  P<0.001 | -3  -4  -2  -1 | 33%  37%  25%  15% | 29%– 37%  33%–40%  23%– 27%  12%– 18% |
| **Clicks per train** |  |  |  |  |
| November  December  January  March | P<0.001  P<0.001  P<0.001  P<0.001 | -3  -3  -4  -1 | 33%  31%  37%  11% | 29%–37%  28%–34%  35%–39%  8%–14% |
| **Modal frequency** |  |  |  |  |
| November  December  January  March | P<0.001  P<0.001  P=0.001  P<0.001 | 3  2  1  -2 | -12%  -7%  3%  8% | -8 %– -17%  -4% – -11%  1%– 5%  5%–11% |
| **Mean SPL** |  |  |  |  |
| November  December  January  March | P<0.001  P<0.001  P<0.001  P=0.3 | -1  -2  -2  0.15 | 14%  19%  19%  -1% | 10%–18%  16%–23%  17%–22%  -4% – +1% |

**Table S3.** The results of the quantile regression models for all acoustic responses are presented below for the reference site (Kahalgaon). The table lists only the tau values that show significant results.

| **Acoustic response** | | **Estimate** | **Standard error** | **Lower**  **CI** | **Upper**  **CI** | **p-value** |
| --- | --- | --- | --- | --- | --- | --- |
| **Train duration** | |  | | | | |
| Tau = 0.9 | Intercept | 0.51 | 0.07 | 1.12 | 1.68 | p<0.001 |
|  | Slope | 0.25 | 0.09 | 0.06 | 0.42 | p=0.009 |
| **Frequency range** | |  | | | | |
| Tau = 0.5 | Intercept | 0.38 | 0.14 | 0.23 | 0.55 | p<0.001 |
|  | Slope | 0.35 | 0.12 | 0.09 | 0.44 | p=0.005 |
| Tau = 0.6 | Intercept | 0.5 | 0.14 | 0.37 | 0.88 | p<0.001 |
|  | Slope | 0.37 | 0.12 | 0.16 | 0.53 | p=0.004 |
| Tau = 0.8 | Intercept | 1.18 | 0.18 | 1.03 | 1.62 | p<0.001 |
|  | Slope | 0.44 | 0.16 | 0.13 | 0.65 | p=0.008 |
| **Clicks per train** | |  | | | | |
| Tau = 0.9 | Intercept | 0.55 | 0.04 | 0.52 | 0.71 | p<0.001 |
|  | Slope | 0.07 | 0.03 | 0.01 | 0.22 | p=0.07 |
| **Modal frequency** | |  | | | | |
| Tau = 0.1 | Intercept | -0.1 | 0.01 | -0.13 | -0.07 | p<0.001 |
|  | Slope | -0.02 | 0.01 | -0.05 | -0.01 | p=0.02 |
| Tau = 0.9 | Intercept | 0.13 | 0.01 | 1.11 | 1.16 | p<0.001 |
|  | Slope | -0.02 | 0.008 | -0.03 | -0.01 | p=0.002 |
| **Mean SPL** | |  | | | | |
| Tau = 0.8 | Intercept | 0.01 | 0.002 | 0.01 | 0.02 | p<0.001 |
|  | Slope | 0.005 | 0.002 | 0.0001 | 0.008 | p=0.03 |
| Tau = 0.9 | Intercept | 0.02 | 0.002 | 0.021 | 0.028 | p<0.001 |
|  | Slope | 0.005 | 0.002 | 0.001 | 0.007 | p=0.02 |

**Supplementary Material S6.**

Table S4. The results for the degree of difference (Dunn’s test) for pair-wise comparisons across sites are presented below. The site-site comparison column shows the difference in acoustic response variables between site-pairs. For the Dunn’s test, the degree (ranks) of difference is given for ‘site—site’ comparisons. Kahalgaon refers to the ‘deep-noisy’ site, Barari to the ‘shallow-quiet’, and Doriganj to the ‘shallow-noisy’ site.

| **Acoustic response** | **Vessel movement** | **Site-site comparison** | **Estimate**  **(ranks of difference)** | **p-value** |
| --- | --- | --- | --- | --- |
| Train duration | Pre | Barari < Doriganj | 10.19 | P<0.001 |
|  |  | Barari > Kahalgaon | 18.85 | P<0.001 |
|  |  | Doriganj > Kahalgaon | 23.02 | P<0.001 |
|  | During | Barari < Doriganj | 12.19 | P<0.001 |
|  |  | Barari > Kahalgaon | 5.29 | P<0.001 |
|  |  | Doriganj > Kahalgaon | 27.85 | P<0.001 |
| Frequency range | Pre | Barari < Doriganj | 18.24 | P<0.001 |
|  |  | Barari > Kahalgaon | 26.96 | P<0.001 |
|  |  | Doriganj > Kahalgaon | 37.08 | P<0.001 |
|  | During | Barari < Doriganj | 17.78 | P<0.001 |
|  |  | Barari > Kahalgaon | 10.51 | P<0.001 |
|  |  | Doriganj > Kahalgaon | 44.6 | P<0.001 |
| Clicks per train | Pre | Barari < Doriganj | 15.71 | P<0.001 |
|  |  | Barari > Kahalgaon | 5.58 | P<0.001 |
|  |  | Doriganj > Kahalgaon | 21.2 | P<0.001 |
|  | During | Barari < Doriganj | 16.82 | P<0.001 |
|  |  | Barari > Kahalgaon | 0.79 | P<0.001 |
|  |  | Doriganj > Kahalgaon | 26.91 | P<0.001 |
| Modal frequency | Pre | Barari > Doriganj | 2.12 | P<0.001 |
|  |  | Barari < Kahalgaon | 14.43 | P<0.001 |
|  |  | Doriganj < Kahalgaon | 11.18 | P<0.001 |
|  | During | Barari > Doriganj | 4.75 | P<0.001 |
|  |  | Barari < Kahlagon | 5.12 | P<0.001 |
|  |  | Doriganj < Kahalgaon | 15.21 | P<0.001 |
| Mean SPL | Pre | Barari < Doriganj | 19.43 | P<0.001 |
|  |  | Barari < Kahalgon | 3.55 | P<0.001 |
|  |  | Doriganj > Kahalgaon | 23.02 | P<0.001 |
|  | During | Barari < Doriganj | 24.69 | P<0.001 |
|  |  | Barari < Kahalgon | 9.93 | P<0.001 |
|  |  | Doriganj > Kahalgaon | 27.04 | P<0.001 |

**Table S5.** The results of the Mann-Whitney test and effect size estimates (Cliff’s delta values) are given in the following table along with the p-value for the Mann-Whitney test and the confidence interval for the Cliff’s delta. A positive estimate for the effect size indicates a percentage increase in acoustic response in the ‘during’ stage compared to the ‘pre’ stage. A negative percentage indicates the opposite trend. Kahalgaon refers to the “deep-noisy” site, Barari to the ‘shallow-quiet’, and Doriganj to the ‘shallow-noisy’ site.

| **Acoustic response**  **(Pre vs. during phases at each site)** | **p-value** | **Estimated mean rank difference** | **Effect size (percentage change from baseline levels)** | **Confidence Interval (Cliff’s Delta 95% C.I.)** |
| --- | --- | --- | --- | --- |
| **Train duration** |  |  |  |  |
| Kahalgaon  Barari  Doriganj | P<0.001  P=0.01  P<0.001 | -45  -20  -66 | 16%  6%  13% | 15%– 18%  0.8%– 11%  8%-18% |
| **Clicks per train** |  |  |  |  |
| Kahalgaon  Barari  Doriganj | P<0.001  P<0.001  P<0.001 | -2  -1  -3 | 26%  16%  21% | 25%– 28%  11%– 21%  16%– 26% |
| **Frequency range** |  |  |  |  |
| Kahalgaon  Barari  Doriganj | P<0.001  P<0.001  P<0.001 | -2  -3  -8 | 23%  19%  32% | 22%– 25%  14%– 24%  28%– 37% |
| **Modal frequency** |  |  |  |  |
| Kahalgaon  Barari  Doriganj | P<0.001  P=0.01  P=0.21 | -1  -2  -2 | 4%  6%  3% | 2%–5%  0.7%–12%  2% – 8% |
| **Mean SPL** |  |  |  |  |
| Kahalgaon  Barari  Doriganj | P<0.001  P=0.05  P<0.001 | -1  0.5  -2 | 14%  -4%  21% | 13%– 15%  -9%– -0.1%  16%– 25% |

**Supplementary Material S7.**

*Shallow water propagation model*

The shallow water propagation model developed by (1) was used to estimate the sound source level (in dB) of the various vessels plying on the river. The equation used for estimating the sound source level was based on the distance of the vessel (*r*) from the recording area and its relation with the depth *H,* at the recording site. A variable *L* is listed in the equation and is related to the layer depth, which is characterised by differing salinity and pressure gradients. Since this condition does not exist in floodplain rivers, the value of *L* was assumed to be the same as the depth *H*. In addition to the distance and depth measurement, factors such as the near-field anomaly and attenuation factor were required to calculate the sound source level. The near-field anomaly *k*_L_, and the attenuation factor$\alpha$_T_, depended primarily on the sea-state, substrate type and the frequency of sound. The values were gathered from data collected by (2). Although these measurements were made in oceanic conditions near the coast, we selected values that closely matched the conditions in the Ganga River. Values for the near-field anomaly k_L_ and attenuation factor$\alpha$_T_ were similar to sea-state 1. The transmission loss (in dB/m) for long ranges (r >*H*) is similar to the cylindrical spreading loss while for short ranges (r <*H*), is similar to spherical spreading loss. However, the equations also account for repeated surface and bottom reflection.

When *r* is less than H, the equation for shallow water propagation is

$$TL=20\log r+\alpha r+60-k_{L}$$

At intermediate ranges where H ≤ *r* ≤ 8H, the equation is

$$TL=15\log r+\alpha r+\alpha_{T}\left( \frac{r}{H}-1 \right)+5\log H+60-k_{L}$$

For longer ranges where *r*>8H, the equation is

$$TL=10\log r+\alpha r+\alpha_{T}\left( \frac{r}{H}-10 \right)+10\log H+64.5-k_{L}$$

*Estimating masking range*

The estimation of masking range requires information on source levels of vessel types at various frequencies along with the source levels of dolphin clicks at corresponding frequencies and the absorption rate, or α in the habitat (4). The absorption rate which was used for the masking range modelling varied slightly from how previous absorption rates were measured. The absorption rate for the masking range consisted of average values of temperature, salinity, depth and pH collected across all sites, with temperature=22^o^C, salinity=0.23 ppt (parts per thousand), depth=9 m, pH=8.48.

The masking range was modelled under the assumptions of a spherical loss model, where we estimated the distance at which the source level of the dolphin clicks would decay and fall to the levels of different motorised vessel noise. This meant that if the SPL of a dolphin click at a particular frequency was below the noise levels of vessel at the same frequency, then the noise from the vessel would completely mask the dolphin click, at that frequency. Therefore, a ‘receiver’ dolphin would not be able to hear the clicks of a ‘transmitter’ dolphin and hence, the ‘transmitter’ dolphin would either have to increase its source level or both the dolphins would need to be closer to each other. This model assumes that the ‘transmitter’ dolphin is located within 10 meters of a vessel. We assumed 10m only as a reference distance for modelling the masking range. This choice was to avoid estimation errors at larger distances and to still represent field observations in which dolphins were often observed in very close proximity to passing vessels. The results were then visualised in the form of a graph to depict the masking ranges due to different vessels in the river.

Figure S3. The spectrogram of a single echolocation click is depicted in the figure below. The colour hues describe the frequency that contains the highest energy and as has been described by several authors, the peak frequency of the dolphin lie around 70 KHz. This individual click was used to estimate the distance a dolphin was at which in turn contributed to the analysis of the masking range. The amplitude scale represents the value from the reference level.


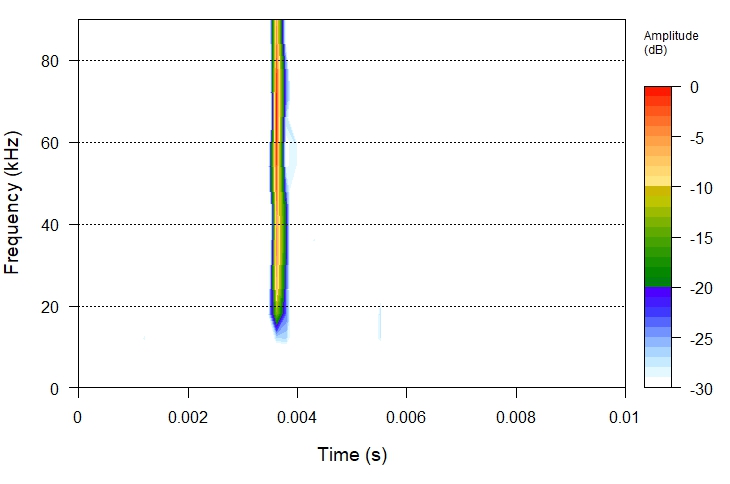


Figure S4. Spectrogram showing the echolocation clicks of a Ganges river dolphin (a). The amplitude scale indicates the relative power level of a click given a reference level. This recording was conducted in a relatively quiet stretch with little to no motorised vessel movement in the area. Spectrogram in (b) when a motorised vessel passes. The cloud of noise that is evident up to 40 kHz and becomes weaker beyond is contributed by cavitation noise. As one can see, cavitation in this high-frequency range interferes with the acoustic signalling of Ganges river dolphin.


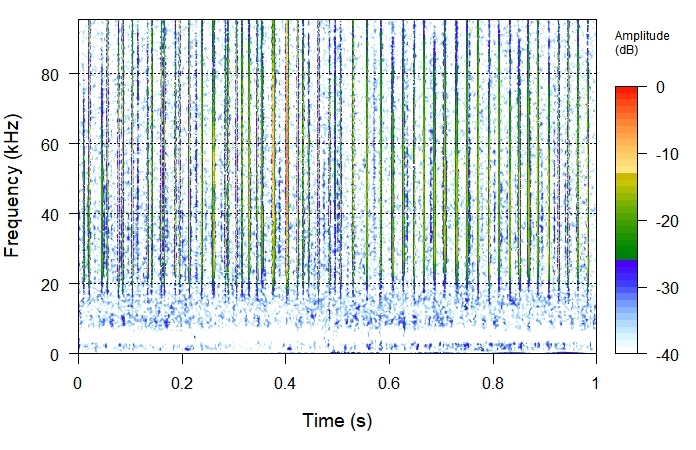


**
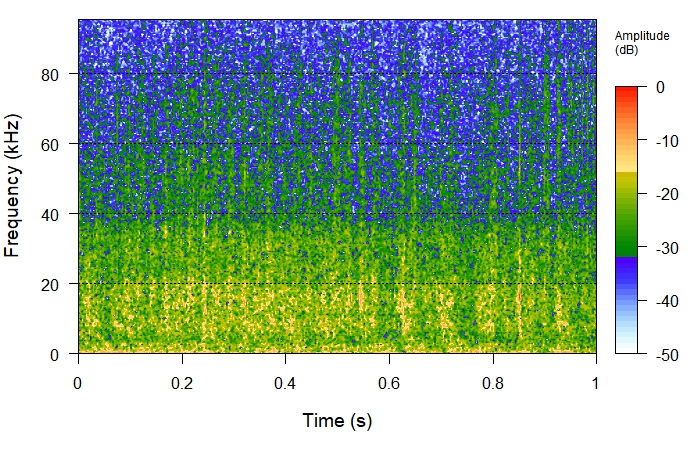
**

*Details of model parameters and variables used in estimation of metabolic costs*

Regression tree models (3) were used to identify ‘rules’ to estimate the ambient noise levels at which significant changes in acoustic responses were observed. These noise levels could be understood as thresholds that led to significant increases in acoustic activity and associated metabolic costs. Figure S6 shows the levels of ambient noise influencing acoustic responses of Ganges river dolphins.

Figure S5 (a–f).The results of the regression tree models that identify points where a change in the ambient noise level leads to significant changes in the acoustic response; (a) Train duration; (b) Clicks per train; (c) Clicks per second; (d) Frequency range; (e) Modal frequency; (f) Average SPL. The values are interpreted as times change from baseline level of no vessel movement. Asterisks indicate levels of statistical significance of the “leaves” of the regression tree models (α = 0.05). All trees were pruned to avoid model overfitting.

**
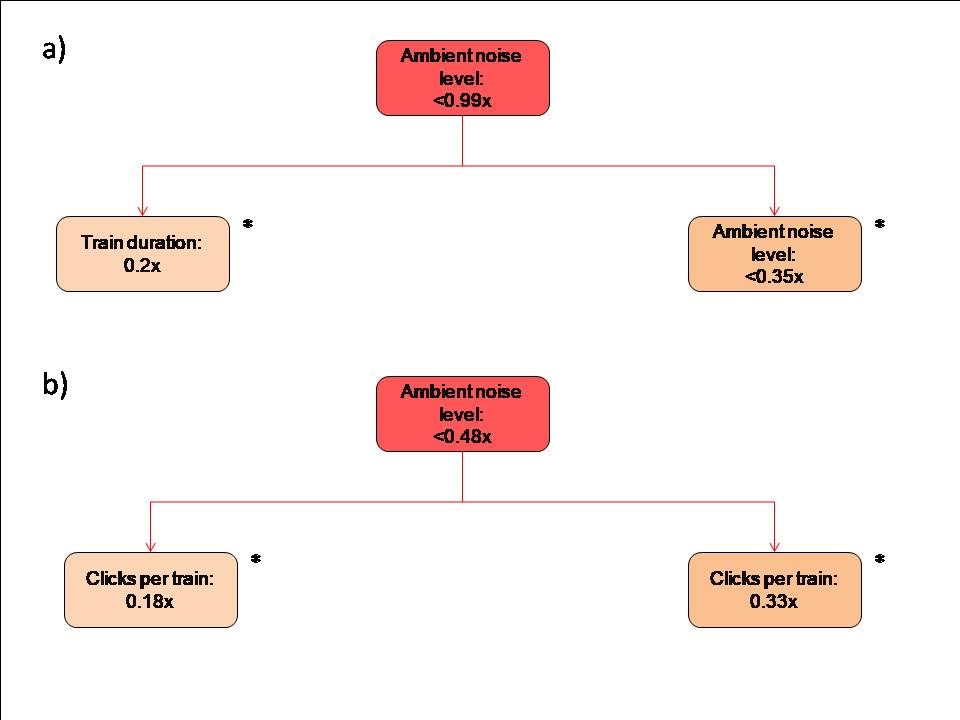
**

**
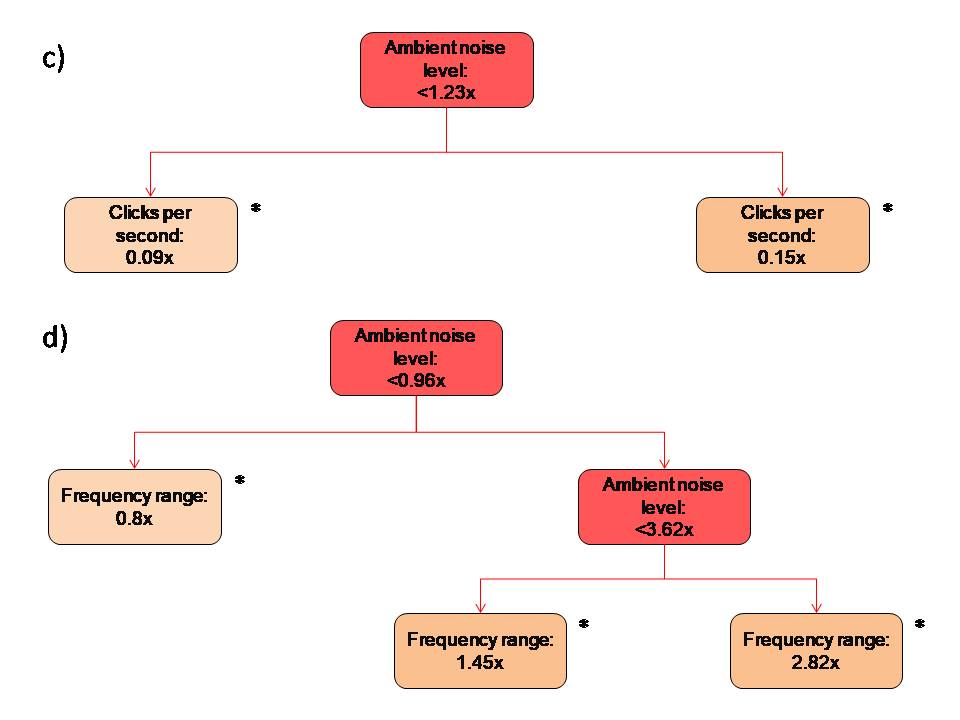
**

**
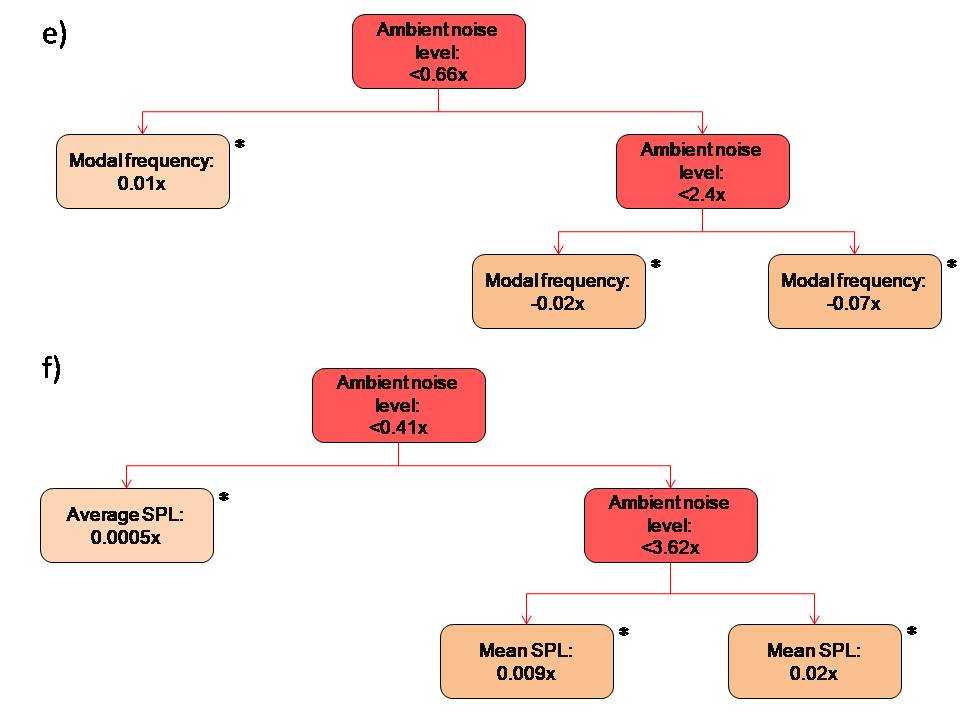
**

**References**

1. Marsh, H. W., & Schulkin, M. Shallow-water transmission. *The Journal of the Acoustical Society of America*, *34*(6), 863-864 (1962).
2. Urick, R. J. The noise background of the sea: ambient noise level. *Principles of Underwater Sound (ed.* Urick, R. J.*)*, 202-236 (1983).
3. De'ath, G., & Fabricius, K.E. Classification and regression trees: a powerful yet simple technique for ecological data analysis. *Ecology*, 81, 3178-3192 (2000).
4. Erbe, C., & Farmer, D. M. Masked hearing thresholds of a beluga whale (*Delphinapterus leucas*) in icebreaker noise. *Deep Sea Research Part II: Topical Studies in Oceanography*, *45*(7), 1373-1388 (1998).
